# Oral Yak Whey Protein Can Alleviate UV-Induced Skin Photoaging and Modulate Gut Microbiota Composition

**DOI:** 10.3390/foods13162621

**Published:** 2024-08-21

**Authors:** Diandian Wang, Yaxi Zhou, Jian Zhao, Chao Ren, Wenjie Yan

**Affiliations:** 1 College of Biochemical Engineering, Beijing Union University, Beijing 100023, China; spwdd2018@163.com (D.W.); 15239407080@163.com (Y.Z.); 2 Beijing Key Laboratory of Bioactive Substances and Functional Food, Beijing Union University, Beijing 100023, China; 18810747451@163.com (J.Z.); rcrc0413@126.com (C.R.)

**Keywords:** yak whey protein, ultraviolet, photoaging, gut microbiota

## Abstract

Excessive UV exposure can lead to skin roughness, wrinkles, pigmentation, and reduced elasticity, with severe cases potentially causing skin cancer. Nowadays, various anti-photoaging strategies have been developed to maintain skin health. Among them, dietary supplements with anti-photoaging properties are gaining increasing attention. Yak whey protein (YWP) possesses multiple benefits, including anti-inflammatory, antioxidant, and immune-boosting properties, effectively protecting the skin. This study used a mixed UVA and UVB light source to irradiate a nude mouse model, exploring the advantages of YWP in anti-photoaging and regulating gut microbiota. The results indicated that YWP alleviated UV-induced skin damage, wrinkles, dryness, and reduced elasticity by inhibiting oxidative stress, inflammatory factors (IL-1α, IL-6, and TNF-α), and matrix metalloproteinases (MMP-1, MMP-3, and MMP-12), thereby increasing the levels of elastin, type I collagen, and type III collagen in the extracellular matrix (ECM). Additionally, YWP significantly improved the abundance of *Firmicutes* and *Bacteroidota* in the gut microbiota of mice, promoting the growth of beneficial bacteria such as *Lachnospiraceae_NK4A136_group*, *Ruminococcus_torques_group*, and *Clostridia_UCG_014*, mitigating the dysbiosis caused by photoaging. These findings underscore the potential of YWP in anti-photoaging and gut microbiota improvement, highlighting it as a promising functional food for enhancing skin and gut health.

## 1. Introduction

The skin is the largest organ of the human body, serving functions such as regulating body temperature, protecting tissues, and shielding against external stimuli like bacteria, viruses, ultraviolet (UV) radiation, and air pollution [1]. Skin aging is divided into intrinsic and extrinsic types [2]. Intrinsic aging, which inevitably results in wrinkles, age spots, sagging, and reduced elasticity, is a natural process that occurs as we age [3]. Extrinsic aging arises from environmental influences. The most significant contributor to this process is extended UV radiation (UVR) exposure, which is commonly known as photoaging. [4]. Based on wavelength, UVR is divided into UVA (320–400 nm), UVB (280–320 nm), and UVC (100–280 nm), with both UVA and UVB being capable of penetrating and damaging the skin [5]. Extensive research has shown that excessive UV exposure leads to an overaccumulation of reactive oxygen species (ROS) within the skin, disrupting the body’s antioxidant system and triggering oxidative stress and inflammatory responses [6]. Furthermore, UVR exposure elevates matrix metalloproteinases (MMPs) in the skin, which in turn accelerates the degradation of collagen, elastin, and the extracellular matrix (ECM). This process intensifies the severity of wrinkles, pigmentation, and loss of skin elasticity beyond what is typically observed with intrinsic aging alone [7,8]. Therefore, boosting the antioxidant defenses of mouse skin while simultaneously inhibiting inflammatory factors and reducing MMP levels are recognized as effective strategies to combat photoaging [9,10].

The skin microbiota and gut microbiota play crucial roles in skin health. Extensive research has confirmed that a complex relationship exists between skin microbiota and skin cells, and the stability of the skin microbiota helps maintain the health of the skin barrier [11,12]. The gut microbiota significantly influences distal organs such as the brain, liver, and skin, regulating immune responses, inflammation, and mental health conditions [13,14,15]. Alterations in the gut microbiota have been connected to the onset of several skin conditions, including psoriasis, atopic dermatitis, and acne [16,17,18]. Currently, modulating the gut microbiota and maintaining skin homeostasis through oral or topical probiotics has become an important approach [19,20]. Additionally, certain proteins and peptides have been shown to regulate the gut microbiota and improve skin photoaging. For example, research by Song et al. revealed that UVB exposure reduced the relative abundance of short-chain fatty acids (SCFAs), *Limosilactobacillus*, *Akkermansia*, *Bacteroides*, and *Ruminococcus* in the gut of mice. However, supplementation with fish collagen significantly increased their relative abundance in the gut microbiota and improved UVB-induced photoaging of the skin in mice [21].

In recent years, various methods have been developed to combat skin photoaging, including specialized cosmetic techniques, the use of cosmeceuticals, and the supplementation of dietary active substances [22,23,24]. Among these, supplementing the body with dietary active substances to fight photoaging and maintain skin health has been a major focus for researchers [25]. Whey protein (WP) is a high-quality dietary protein source known for its benefits in enhancing immunity, fighting cancer, managing diabetes, regulating the gut microbiota, and protecting the skin [26]. Early studies found that oral administration of whey protein could alleviate UVB-induced collagen type IV degradation and DNA damage in mouse skin, while also inhibiting the expression of MMP-9, thereby improving skin wrinkles and elasticity loss in photoaged mice [27]. Further research indicated that the effective anti-photoaging components of whey protein might be derived from β-lactoglobulin and α-lactalbumin [28]. Yak whey protein (YWP) contains higher levels of β-lactoglobulin and α-lactalbumin compared to whey proteins from cow’s and goat’s milk [29]. However, there is currently a lack of research on YWP’s effects on photoaging and gut microbiota regulation. Therefore, this study explores the impact of YWP on UV-induced skin photoaging and intestinal flora imbalance in mice, addressing the gap in research on YWP in this area.

## 2. Materials and Methods

### 2.1. Materials

Yak milk was purchased from Hongyuan Yak Dairy Co., Ltd. (Chengdu, China). Superoxide dismutase (SOD) and malondialdehyde (MDA) assay kits were obtained from Nanjing Jiancheng Bioengineering Institute (Nanjing, China). The BCA protein concentration assay kit was purchased from Beijing Biotech Co., Ltd. (Beijing, China). β-actin, MMP-1, 3, 12, and elastin antibodies were acquired from Jiangsu Affinity Biosciences Research Center Co., Ltd. (Changzhou, China). The Trizol reagent kit was purchased from Aidlab Biotechnologies Co., Ltd. (Beijing, China). The reverse transcription kit ExonScript RT SuperMix with dsDNase was procured from Chengdu Exongen Biotechnology Co., Ltd. (Chengdu, China).

### 2.2. Yak Whey Protein (YWP) Preparation

The method for preparing yak whey protein was adapted from previous research with appropriate modifications [30]. First, the frozen yak milk was thawed at room temperature. The milk was then centrifuged at 10,000 r/min for 20 min to remove fat, discarding the top layer to obtain skim milk. The pH of the skim milk was regulated to 4.6 using hydrochloric acid and sodium hydroxide to precipitate casein. Following the pH regulation, the mixture was subjected to centrifugation at 4500 r/min for 20 min. The supernatant obtained after centrifugation was then collected and designated as the raw yak whey liquid. This liquid was then dialyzed using a regenerated cellulose membrane, which has a molecular weight cut-off range of 12,000–14,000 Da. After 24 h, the retained liquid was collected and subjected to vacuum freeze-drying to obtain yak whey protein powder. The lyophilized powder was preserved at −20 °C for subsequent use. Further, the free and total amino acid contents of YWP were determined using a high-speed amino acid analyzer (LA8080) (Hitachi High-tech International Trade Co., Ltd., Shanghai, China) according to a previously established method [31]. The results of the determination are shown in Table 1.

### 2.3. Animal Model Construction

All animal-related experiments were conducted in compliance with the “Regulations on Animal Management” and followed the relevant guidelines of the Animal Welfare and Ethics Committee of Beijing Union University (Approval No.: JCZX11-2402-2). Forty 6-week-old female SPF-grade nude mice, weighing 18–20 g (License No.: SCXK(Beijing)2019-0008), were obtained from Beijing Huafukang Bioscience Co., Ltd. (Beijing, China). They were first acclimated for one week in a sterile, temperature-controlled SPF-grade animal facility (22 °C, 50% humidity) with free access to food and water and a 12 h light/dark cycle. The mice were then randomly divided into five groups, with eight mice in each group: the normal control group (NC, sterile water), the UVR group (UVR, sterile water), the low-dose YWP group (YWP-L, 200 mg/kg·bw), the medium-dose YWP group (YWP-M, 400 mg/kg·bw), and the high-dose YWP group (YWP-H, 800 mg/kg·bw).

**Figure 1:**
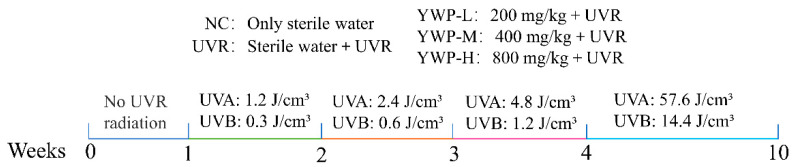
Experimental protocol of ultraviolet irradiation and gavage in mice.

Except for the blank control group, the groups were exposed to UVR starting from the second week. The exposure regimen was as follows: 10 min per day in the second week, 20 min per day in the third week, 40 min per day in the fourth week, and 80 min per day from the fifth to the tenth week. The UV exposure was carried out using two UVA lamps (wavelength range 315–400 nm, 40 W) and one UVB lamp (wavelength range 290–315 nm, 40 W), purchased from Shenzhen Guanyu Photonics Technology Co., Ltd. (Shenzhen, China). The lamps were placed 35–40 cm above the mice’s backs, and exposure began 2 h after daily gavage. The irradiation intensity was measured using an LS125 multi-probe UV radiometer (Shenzhen Linshang Technology Co., Ltd., Shenzhen, China), with UVA and UVB intensities at 0.4 mW/cm² and 0.1 mW/cm², respectively. The irradiation dose (mJ/cm²) was calculated as irradiation intensity (mW/cm²) × irradiation time (s). The weekly irradiation doses are shown in Figure 1. The total UV doses received by the mice’s backs were 66 J/cm² for UVA and 16.5 J/cm² for UVB. After the final UV exposure, mouse feces were collected, the mice were euthanized by cervical dislocation, and the skin from their backs was quickly collected.

### 2.4. HE Staining

The mouse skin tissues were immersed in a tissue fixative solution and left to fix for a duration of 24 h. Following fixation, the tissues were dehydrated, embedded, and sectioned, followed by staining with hematoxylin and eosin (HE). The structure of the skin tissues was then examined under a biological microscope at 100× and 400× magnifications.

### 2.5. Biochemical Detection

The skin tissues were homogenized with nine times their volume of saline in an ice-water bath. The contents of SOD and MDA in the skin tissues were then measured using kits produced by Nanjing Jiancheng Bioengineering Institute (Nanjing, China). The experimental procedures strictly followed the guidelines provided in the kit instructions.

### 2.6. Western Blotting Analysis

The levels of MMP-1, 3, 12, and elastin were detected using Western blotting. This method was adapted with modifications from Song et al. [32]. First, the tissues were thawed and homogenized, followed by cell disruption using ultrasonic treatment in an ice bath. Following centrifugation, the supernatant was carefully collected, and the protein concentration was quantified using the BCA protein assay method.

Subsequently, proteins were resolved by sodium dodecyl sulfate–polyacrylamide gel electrophoresis (SDS-PAGE) and transferred onto a PVDF membrane. The membrane was then blocked in a blocking solution for 1 h, followed by an overnight incubation with primary antibodies at 4 °C. After being washed with TBST, the PVDF membrane was incubated with secondary antibodies for 1 h at room temperature in a dark environment, and then the membrane was washed again with TBST to remove any unbound secondary antibodies.

Finally, the membrane was visualized using ECL protein detection reagents, photographed with a chemiluminescence imaging system ChemiScope 6100 (Shanghai Qinxiang Scientific Instrument Co., Ltd., Shanghai, China), and analyzed using ImageJ software (V 1.54j). The protein expression levels were normalized to β-actin, which was used as an internal control.

### 2.7. Real-Time Fluorescence Quantitative PCR (RT-qPCR)

The respective RNA levels of collagen I (*COL1A1*), collagen III (*COL3A1*), and inflammatory factors IL-1α, IL-6, and TNF-α in the skin tissues were quantified through RT-qPCR analysis. Total RNA was isolated from the skin tissues using the Trizol method, and RNA concentration and purity were measured using a nucleic acid concentration analyzer. The reaction system was established according to the instructions of the reverse transcription kit, and cDNA was synthesized via reverse transcription on a PCR instrument. The target genes were then amplified, and the relative expression levels of the target genes were analyzed using the 2^−△△Ct^ method with β-actin as the internal reference gene. The primer sequences are shown in Table 2.

### 2.8. 16S rRNA Sequencing

We commissioned Shanghai Applied Protein Technology Co., Ltd. (Shanghai, China) to perform 16S rRNA gene sequencing on mouse intestinal contents, following a modified protocol [33]. DNA was extracted using the EZNA Stool DNA Kit (Omega Bio-tek, Norcross, GA, USA), and its concentration and purity were assessed on a 1% agarose gel. The DNA was then diluted to 1 ng/μL, and PCR amplification targeted the V3-V4 region using universal primers 338F (5′-ACTCCTACGGGAGGCAGCA-3′) and 806R (5′-GGACTACHVGGGTWTCTAAT-3′). Sequencing was conducted on the Illumina NovaSeq 6000 platform. Chimeric sequences were filtered using Versaeach software (V 2.3.4), and ASVs were annotated with the RDP algorithm against the Silva 16S rRNA database at a 70% confidence threshold. Data analysis included LEfSe, α-diversity, and β-diversity analyses, performed on the Microbiome Analyst platform (https://www.microbiomeanalyst.ca, 15 June 2024) [34].

### 2.9. Statistical Analysis

Statistical analyses were conducted using IBM SPSS Statistics 27.0 software. Results are expressed as mean ± SD. One-way analysis of variance (ANOVA) followed by Tukey’s post hoc test was utilized to assess the significance of differences between experimental groups. Statistical significance is indicated by distinct lowercase letters at the *p* < 0.05 level.

## 3. Results

### 3.1. Macroscopic and Histologic Effects of YWP on Mouse Skin

**Figure 2:**
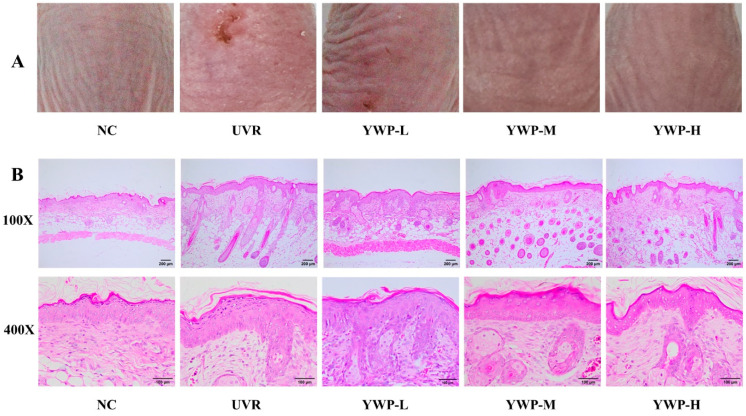
Macroscopic and histologic effects of YWP on mouse skin. (**A**) Representative photographs of mouse dorsal skin. (**B**) HE staining results.

As shown in Figure 2A, the back skin of mice in the NC group displayed only fine wrinkles, which are considered normal skin textures, and the skin was elastic without dryness or peeling. After UVR exposure, the mice’s skin exhibited severe wounds and erythema, along with visible dryness, peeling, and deep wrinkle furrows. This indicates that the combined UVA and UVB light source can induce photoaging in the mice’s skin. Upon administering different doses of YWP to the mice, it was observed that as the dose increased, the features of photoaging gradually diminished, and the symptoms of wounds, wrinkles, and dryness caused by photoaging progressively disappeared. In the YWP-H dose group, the deep wrinkles on the mice’s back skin had almost completely vanished, and no peeling was observed.

Figure 2B presents the HE staining results of the mouse skin tissues. UVR exposure dramatically increased the thickness of the mouse epidermis, with noticeable thickening in the stratum corneum and stratum spinosum. Additionally, in the UVR group, there were evident disruptions and disorganization in the collagen and elastic fibers within the dermal tissue. However, relative to the UVR group, the YWP-M group exhibited a thinner epidermis, a deeper coloration, a more orderly arrangement of dermal fibers, and a fuller morphology of sebaceous glands and hair follicles, indicating improvements in the features of photoaging. In the YWP-H group, these beneficial effects were even more pronounced. This suggests that oral administration of YWP can significantly improve the morphological changes in the skin induced by UVR.

### 3.2. Effect of YWP on Oxidative Stress and Inflammation Levels in Mouse Skin

**Figure 3:**
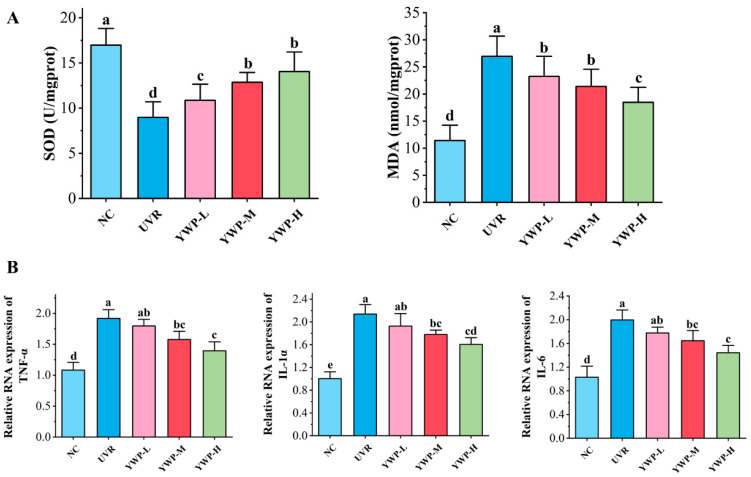
Effect of YWP on oxidative stress and inflammation levels in mouse skin. (**A**) SOD and MDA content in mouse skin (*n* = 6). (**B**) mRNA expression of TNF-α, IL-1α, and IL-6d in mouse skin tissue (*n* = 6). Statistical significance is indicated by distinct lowercase letters at the *p* < 0.05 level.

As shown in Figure 3A, the UVR group dramatically reduced the content of the antioxidant enzyme SOD in mouse skin tissues and increased the levels of the oxidative product MDA. However, low doses of YWP significantly decreased MDA levels and increased SOD levels. YWP showed a good dose-dependent effect on enhancing the skin’s antioxidant capacity. Additionally, RT-qPCR analysis was conducted to quantify the levels of inflammatory factors in mouse skin tissues, and the findings are displayed in Figure 3B. In the NC group, the RNA levels of IL-1α, IL-6, and TNF-α were maintained at low levels. After UVR exposure, the release of inflammatory factors was significantly promoted, leading to a sharp increase in the RNA levels of IL-1α, IL-6, and TNF-α. However, YWP-M and YWP-H significantly reduced the production of UVR-induced inflammatory factors, showing a certain dose-dependent effect.

### 3.3. Effect of YWP on MMPs, Elastin, and Collagen in Mouse Skin

**Figure 4:**
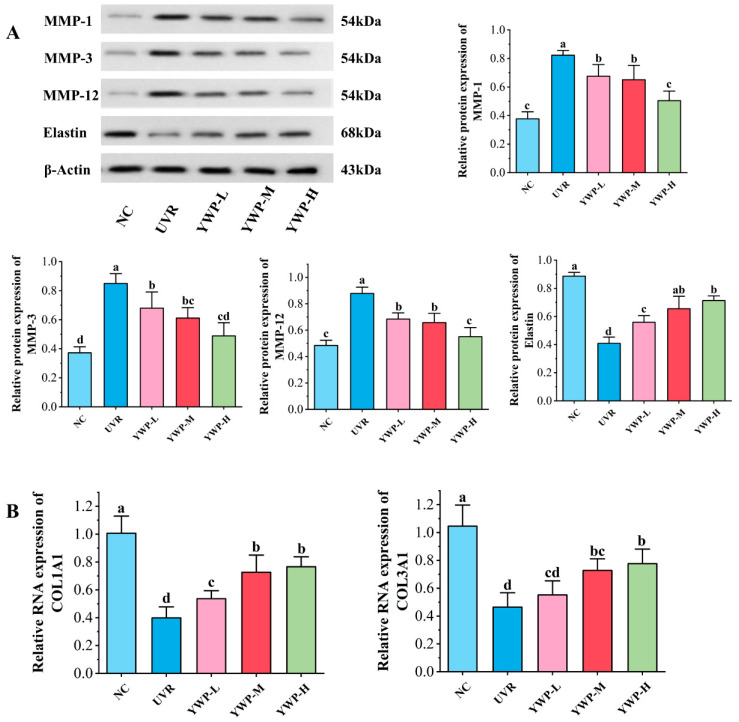
Effect of YWP on MMPs, elastin, and collagen in mouse skin. (**A**) Protein expression of MMP-1, MMP-3, MMP-12, and elastin in mouse skin tissues measured by WB (*n* = 3). (**B**) RT-qPCR of *COL1A1* and *COL3A1* mRNA expression (*n* = 6). Statistical significance is indicated by distinct lowercase letters at the *p* < 0.05 level.

In Figure 4A, UVR exposure led to increased levels of MMP-1, MMP-3, and MMP-12, and induced a reduction in elastin levels in the tissues. Supplementation with low doses of YWP significantly reduced the expression levels of MMPs. While the inhibition of MMPs did not differ significantly between the low- and medium-dose groups, the high-dose group nearly restored MMP expression to the control group levels. Additionally, low doses of YWP significantly increased the content of elastin, while medium doses of YWP restored elastin to levels comparable to the normal group.

Furthermore, as shown in Figure 4B, at the gene level, UVR exposure significantly reduced the RNA expression levels of collagen I and collagen III compared to the NC group. Low doses of YWP significantly reversed the decrease in collagen I RNA levels, and while this dose also increased the expression of collagen III RNA, it was not significant. Medium doses of YWP significantly increased the RNA expression levels of collagen I and collagen III; however, the difference between the medium and high doses was not statistically significant.

### 3.4. Effect of YWP on the Structure of Intestinal Flora in Photoaged Mice

**Figure 5:**
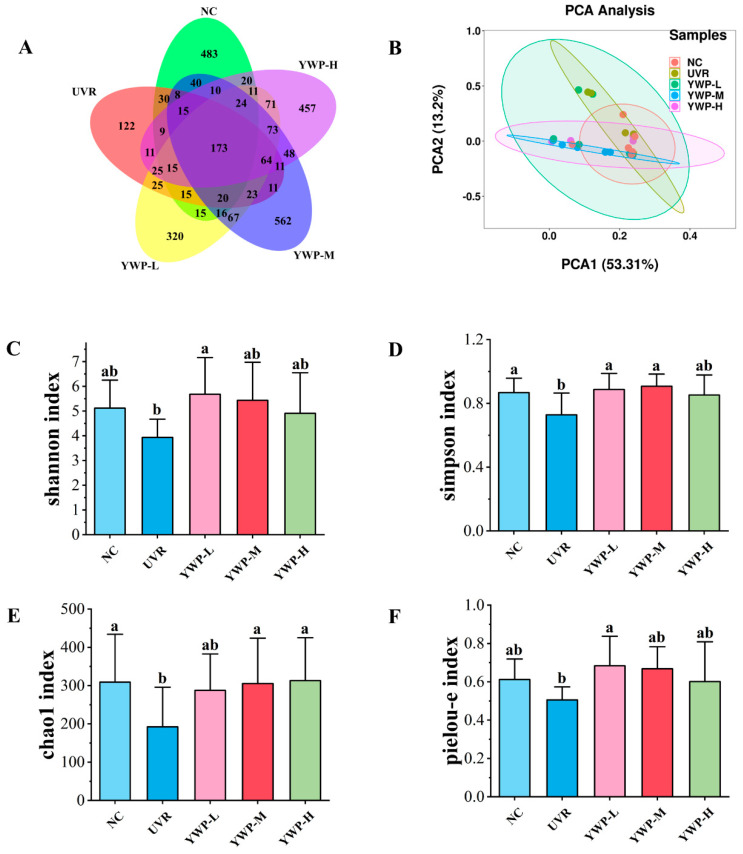
Effect of YWP on the structure of intestinal flora in mice. (**A**) Venn diagram showing the number of ASVs in intestinal microbiota across different groups of mice. (**B**) Beta diversity analysis of intestinal microbiota across different groups of mice. (**C**–**F**) Analysis of the number of ASVs in intestinal microbiota across different groups of mice, including the Shannon index, Simpson index, Chao1 index, and Pielou-e index. Statistical significance is indicated by distinct lowercase letters at the *p* < 0.05 level.

Fecal samples from each group of mice underwent 16S rRNA gene sequencing and analysis, with the findings presented in Figure 5. The Venn diagram (Figure 5A) illustrates the number of shared and unique Amplicon Sequence Variants (ASVs) for each group. The NC group contained 904 ASVs, of which 483 were unique; the UVR group contained 577 ASVs, with 122 being unique; the YWP-L group contained 957 ASVs, with 320 being unique; the YWP-M group contained 1165 ASVs, with 562 being unique; and the YWP-H group contained 1037 ASVs, with 457 being unique. These data indicate that UVR exposure reduced the diversity of the gut microbiota, but supplementation with YWP could restore the diversity of the gut microbiota.

Figure 5C–F show the results of the Alpha diversity analysis, reflecting species richness and evenness among the different groups. It can be seen that UVR exposure led to a decrease in the Shannon, Simpson, Chao1, and Pielou-e indices in the UVR group. However, low doses of YWP significantly increased the Shannon, Simpson, and Pielou-e indices, enhancing the diversity and evenness of the gut microbiota in photoaged mice. The medium dose of YWP significantly increased the Chao1 index, with the recovery level approaching that of the NC group. Principal Component Analysis (PCA) (Figure 5B) further confirmed that the composition of the gut microbiota in mice was influenced by UVR and YWP.

**Figure 6:**
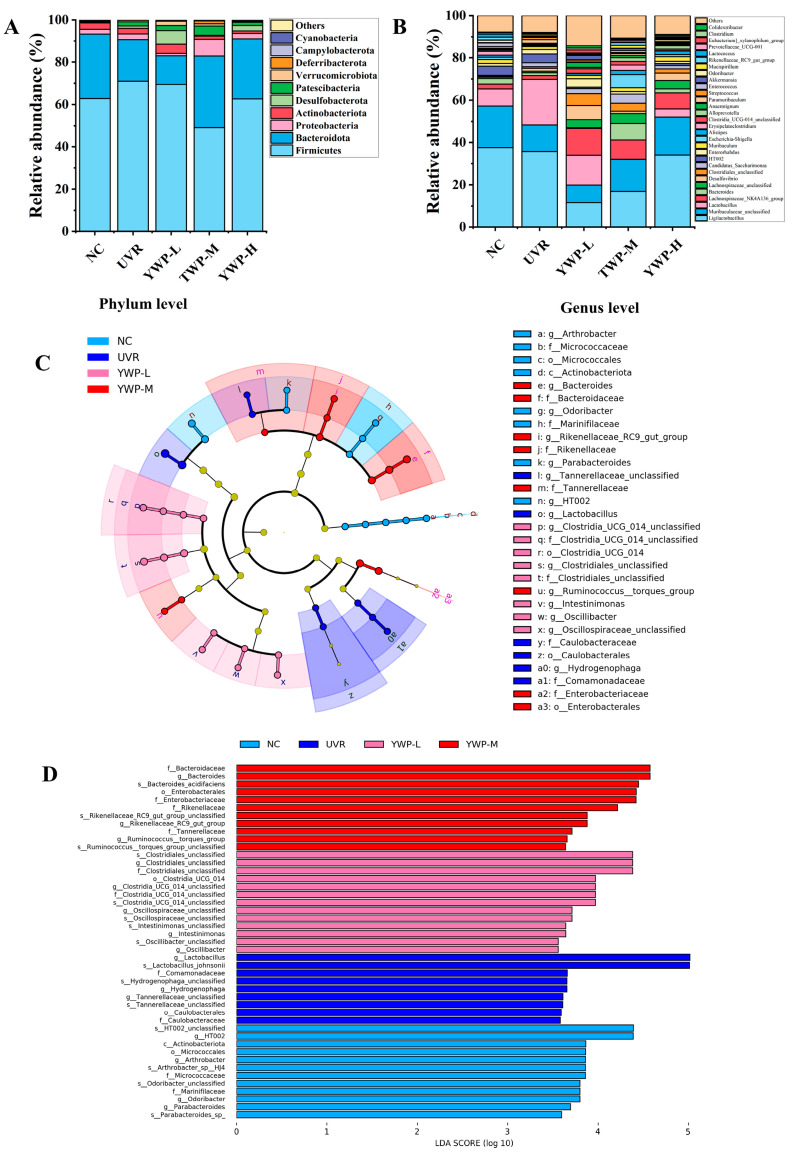
Structure of mouse intestinal flora and results of LEfSe analysis. (**A**) Top 10 relative abundances at the level of mouse intestinal flora phylum in each group. (**B**) Top 30 relative abundances at the level of mouse intestinal flora genus in each group. (**C**) Evolutionary branching diagram of LEfSe analysis. (**D**) Distribution histogram (LDA > 3.5).

Figure 6A shows the relative abundance of the top 10 microbial phyla in the gut microbiota of mice, including *Firmicutes*, *Bacteroidota*, *Proteobacteria*, *Actinobacteriota*, and *Desulfobacterota*. In comparison to the NC group, the UVR group exhibited elevated levels of *Firmicutes* and decreased levels of *Bacteroidota*. Medium doses of YWP significantly reduced the levels of *Firmicutes*. Interestingly, after YWP intervention, the levels of *Desulfobacterota* and *Verrucomicrobiota* also increased to varying degrees. Figure 6B displays the relative abundance of the top 30 microbial genera in the gut microbiota of mice. Notable changes were observed in *Ligilactobacillus*, *Muribaculaceae_unclassified*, Lactobacillus, *Lachnospiraceae_NK4A136_group*, *Lachnospiraceae_unclassified*, *Clostridiales_unclassified*, and *Alistipes*. Compared to the UVR group, YWP intervention increased the species abundance of *Lachnospiraceae_NK4A136_group*, *Lachnospiraceae_unclassified*, *Clostridiales_unclassified*, and *Alistipes*.

LDA Effect Size (LEfSe) analysis was employed to identify biomarkers that showed significant differences between groups, as shown in Figure 6C,D. With the LDA threshold set at 3.5, there were no significantly different species between the YWP-H group and the other groups. However, there were significantly different species among the other four groups. In the NC group, significantly different species included *HT002*, *Actinobacteriota*, and *Arthrobacter*. In the UVR group, significantly different species included *Lactobacillus_johnsonii*, *Comamonadaceae*, *Hydrogenophaga_unclassified*, and *Hydrogenophaga*. In the YWP-L group, significantly different species included *Clostridiales_unclassified* and *Clostridia_UCG_014_unclassified*. In the YWP-M group, significantly different species included *Bacteroidaceae*, *Bacteroides_acidifaciens*, and *Enterobacterales*.

## 4. Discussion

The skin acts as the first line of defense for the organism, playing a crucial role in protecting against external environmental pollution and maintaining internal environmental stability [35]. However, prolonged exposure to UVR can lead to photoaging of the skin. Preventing and delaying photoaging is a major focus of current research [36]. Regulating the microbiota of the gut has proven to be an effective method for improving photoaging [37,38]. In this study, we used a combination of UVA and UVB light sources to induce photoaging in nude mouse skin to explore the potential benefits of YWP in alleviating photoaging and regulating the gut microbiota.

### 4.1. YWP Ameliorates Clinical Signs and Reduces Oxidation and Inflammation Levels in Skin Tissues in Photoaged Mice

Previous reports have shown that UVR radiation can cause a loss of skin barrier function, excessive damage to the ECM, and result in wrinkles, roughness, pigmentation, and significantly reduced skin elasticity [23,39]. In this study, after 10 weeks of UVR exposure, mice exhibited noticeable wounds and erythema on their back skin, along with dry, peeling skin and deep wrinkle furrows (Figure 2A). HE staining results indicated that the UVR group had a thickened epidermis, atrophied sebaceous glands, disorganized collagen and elastic fibers, and a reduced number of these fibers (Figure 2B), demonstrating clear symptoms of skin photoaging.

Furthermore, normal skin tissue maintains a stable redox balance, but excessive UVR induces the production of ROS, disrupting the dynamic balance between oxidation and antioxidation, leading to an inability to promptly eliminate the excess ROS [40]. ROS accumulation in skin tissues damages the activity of antioxidant enzymes, induces the production of lipid peroxides and inflammatory factors, and consequently destroys the bio-membranes and proteins of skin cells [41,42].

In this study, UVR exposure resulted in decreased levels of the antioxidant enzyme SOD and increased levels of the lipid peroxide MDA. Under oxidative stress, the transcription levels of inflammatory factors (IL-1α, IL-6, and TNF-α) in the skin tissues significantly increased compared to the NC group (Figure 3). However, supplementation with YWP significantly improved UVR-induced skin damage. HE results showed that high doses of YWP markedly increased the number of collagen and elastic fibers, with tighter connections between dermal fibers, and alleviated sebaceous gland atrophy and epidermal thickening. Additionally, medium doses of YWP significantly enhanced the antioxidant levels in mice and reduced skin inflammation, indicating the beneficial effects of YWP on photoaged skin.

### 4.2. YWP Promotes Skin Elastin and Collagen Production by Inhibiting the Expression of MMPs

Elastin is the main protein in elastic fibers, providing skin with firm extensibility [43]. Type I and III collagen are the most abundant proteins in the skin dermis, providing tensile strength [44]. *COL1A1* and *COL3A1* are genes that synthesize the α-1 chains of type I and type III collagen, respectively, and are crucial for the synthesis of collagen trimers [45]. Collagen and elastin, key components of the ECM, are essential for maintaining skin elasticity and resilience; their loss directly leads to skin aging and sagging [43]. UVR radiation induces the release of MMPs from dermal fibroblasts and keratinocytes [46]. MMPs are a class of endopeptidases dependent on metal ions like Ca^2+^ or Zn^2+^, capable of degrading almost all components of the ECM, especially collagen and elastin [47]. Among them, MMP-1 degrades type I and type III collagen, MMP-3 cuts the remaining collagen fragments, and MMP-12 is the primary MMP that destroys elastin, playing a significant role in elastin loss due to photoaging [48,49].

In this experiment, WB results showed that UVR exposure significantly increased the levels of MMP-1, MMP-3, and MMP-12, while reducing the expression of elastin (Figure 4A). RT-qPCR results indicated that UVR exposure reduced the transcription of COL1A1 and COL3A1 genes (Figure 4B). These findings demonstrate that UVR exposure leads to the loss of elastin and collagen, which is a primary cause of wrinkle formation in mouse skin. However, in our experiment, low doses of YWP showed significant effects in reducing MMPs and promoting the transcription of elastin, type I collagen, and type III collagen genes, indicating the effective role of YWP in combating skin photoaging. Of course, these improvements are linked to the inhibition and activation of various additional signaling pathways. According to reports, the activated TGF-β/Smad signaling pathway can increase the formation of collagen while inhibiting MMP-1 and MMP-3 in skin tissues [50]. However, the mechanism of how YWP improves photoaged skin is not fully understood. As a result, further work has to be carried out to better understand the mechanism of how YWP prevents photoaging and investigate YWP’s positive impacts on skin health.

### 4.3. YWP Exerts Anti-Photoaging Activity by Promoting the Production of Beneficial Intestinal Bacteria

Most studies have shown that photoaging induces changes in the gut microbiota of mice [51,52]. However, dietary supplementation or topical application of bioactive components can not only improve the photoaging of mice but also increase the richness and number of beneficial bacteria, further enhancing the anti-photoaging effects [21,38]. The richness and diversity of the gut microbiota are important indicators of gut health. Alpha diversity analysis mainly reflects species richness and evenness [53]. Figure 5C,D show the main results of the Alpha diversity analysis. It can be seen that UVR exposure significantly reduced the Simpson index and Chao1 index of the gut microbiota in mice, and also suppressed the Shannon index and Pielou-e index, though the results were not significant. However, after YWP treatment, the diversity of the intestinal microbiota significantly increased. Previous studies have shown that the higher the diversity of the intestinal microbiota, the stronger its adaptability to external factors and its ability to maintain internal balance [54].

The gut microbiota of mice mainly consists of Firmicutes and *Bacteroidota*, but UVR exposure led to increased levels of *Firmicutes* and decreased levels of Bacteroidota in the UVR group, resulting in an increased F/B (*Firmicutes/Bacteroidota*) ratio (Figure 6A). Numerous studies have shown that both excessively high and low F/B ratios are considered signs of gut microbiota imbalance. An excessively high F/B ratio can lead to obesity and metabolic disorders [55,56]. Similarly, in previous studies, excessive UVB exposure on the backs of mice also led to increased levels of *Firmicutes* [57,58]. However, in this study, high doses of YWP significantly restored the F/B ratio, achieving the effect of regulating the intestinal microbiota.

LEfSe analysis results showed significant differences in dominant species between groups. After YWP treatment, the abundance of *Ruminococcus_torques_group* and *Clostridia_UCG_014* in the gut of mice increased. *Clostridia_UCG_014* promotes the growth of beneficial gut bacteria and enhances immune function [59]. *Ruminococcus* is a butyrate-producing bacterium that plays an important role in inhibiting inflammation and promoting short-chain fatty acids [60]. Similarly, treatments with pearl powder and fish collagen also showed an increased abundance of *Ruminococcus* in photoaged mice [21,38]. Therefore, there might be a close link between *Ruminococcus* and the treatment of skin photoaging, which deserves further research and validation. These results suggest that YWP enhanced its anti-photoaging effect by promoting the abundance of intestinal flora and the number of beneficial bacteria, revealing a link between intestinal flora and skin photoaging.

## 5. Conclusions

In summary, our results indicate that daily oral administration of YWP for 10 weeks significantly improved photoaging symptoms and modulated the intestinal flora of mice’s skin induced by a mixed UVA and UVB light source. The therapeutic results of YWP showed some dose dependence, with 800 mg/kg·bw of YWP demonstrating the best ameliorative effect. On one hand, YWP significantly enhanced the antioxidant capacity of the mice’s skin, reduced the transcription levels of inflammatory factors TNF-α, IL-1α, and IL-6, decreased the protein expression of MMP-1, MMP-3, and MMP-12, and increased the expression of elastin, type I collagen, and type III collagen. On the other hand, YWP improved the photoaging of mice’s skin by increasing the richness and number of beneficial bacteria in the gut microbiota, with *Ruminococcus_torques_group* and *Clostridia_UCG_014* being identified as key microbiota for treating photoaging. These results highlight the possibility of YWP as a functional food for improving photoaging, which provides certain directions for the subsequent development of YWP. Meanwhile, this study will enrich the choice of anti-photoaging products and broaden the application of natural dietary proteins in functional foods.

## Figures and Tables

**Table 1:** Amino acid composition and total amino acid content of YWP.

Amino Acid Type	Content (g/100 g)	Amino Acid Type	Content (g/100 g)
aspartic acid (Asp)	9.92	leucine (Leu) *	7.98
threonine (Thr) *	4.24	tyrosine (Tyr)	2.56
serine (Ser)	4.38	phenylalanine (Phe) *	2.92
glutamic acid (Glu)	13.38	lysine (Lys) *	7.24
glycine (Gly)	1.698	histidine (His)	2.14
alanine (Ala)	2.44	arginine (Arg)	1.926
valine (Val) *	3.28	proline (Pro)	3.52
methionine (Met) *	1.178	essential amino acid	30.985
isoleucine (Ile) *	4.12	total amino acid	72.922

The “*” marks the essential amino acids.

**Table 2:** Sequence of primers for RT-qPCR.

Gene Name	Forward Sequence (5′-3′)	Reverse Sequence (5′-3′)
*COL1A1*	GAGAACATCCGCAGCCCCGAAG	CGATCCAGTACTCTCCGCTCT
*COL3A1*	CCCAACCCAGAGATCCCATT	GGTCACCATTTCTCCCAGGA
*IL-1α*	CCGTGTTGCTGAAGGAGTTG	GTGCACCCGACTTTGTTCTT
*IL-6*	ACTTCCATCCAGTTGCC	ATGTGTAATTAAGCCTCCGAC
*TNF-α*	ACGGCATGGATCTCAAAGACAAC	AGATAGCAAATCGGCTGACGG
*β-Actin*	CTCCTGAGCGCAAGTACTCT	TACTCCTGCTTGCTGATCCAC

## Data Availability

The original contributions presented in the study are included in the article, further inquiries can be directed to the corresponding author.
